# Assessment of community health volunteers’ knowledge on cervical cancer in Kadibo Division, Kisumu County: a cross sectional survey

**DOI:** 10.1186/s12913-017-2593-5

**Published:** 2017-09-25

**Authors:** Edwin Onyango Ochomo, Harrysone Atieli, Sussy Gumo, Collins Ouma

**Affiliations:** 1 School of Public Health and Community Development, Maseno University, Private Bag, Maseno, Kenya; 2 School of Arts and Social Sciences, Maseno University, Private Bag, Maseno, Kenya; 3 Ideal Research Center, P.O. Box 7244-40123, Kisumu, Kenya

**Keywords:** Community health volunteers, Knowledge, Cervical cancer

## Abstract

**Background:**

Globally, cervical cancer is the fourth most frequent cancer in women, with an estimated 530,000 new cases in 2012, representing 7.5% of all female cancer deaths. Of the estimated more than 270,000 deaths from cervical cancer every year, more than 85% occur in less developed regions. In sub-Saharan Africa, 34.8 new cases of cervical cancer are diagnosed per 100,000 women annually, and 22.5/100,000 women die from the disease. Despite the magnitude of this problem, Kenya still has a screening rate of 3.2%; therefore, cervical cancer prevalence has not been established. Community Health Volunteers (CHV) are required to create demand for screening in the community and capture this in the Ministry of Health (MOH) reporting tool MOH 514. The objective of this study was to determine the knowledge of risk factors, signs and symptoms of cervical cancer and screening services’ availability amongst CHVs to enable them sensitize the community about cervical cancer in Kadibo Division, Kisumu County.

**Method:**

In a cross-sectional study, a saturated sample of 188 CHVs was interviewed. The knowledge of cervical cancer was presented by use of frequencies and proportions; the relationship between demographic characteristics and knowledge was determined using chi-square.

**Results:**

A majority, 161 (85.6%), were women, 47 (25.0%) were aged 40–44, 91 (48.4%) had primary education and 132 (70.2%) were small-scale farmers. A total of 128 (68.1%) had low, 60 (31.9%) had average and none had high amount of knowledge of risk factors. On average, 95 (50.5%) had low, 15 (8.0%) had average and 78 (41.5%) had high amount of knowledge of signs and symptoms. Finally, 77 (41.0%) had high, 40 (21.2%) had average and 71 (37.8%) had low knowledge of the availability of screening services. Education (*p* = 0.012, χ^2^ = 3.839), occupation (*p* < 0.0001, χ^2^ = 12.722), and health centre of attachment (p < 0.0001, χ^2^ = 71.013) were significant factors in determining the knowledge of risk factors. The knowledge of the signs and symptoms of cervical cancer was determined by the occupation of the CHVs (*p* = 0.030, χ^2^ = 15.110) and the years of work as a CHV (*p* = 0.014, χ^2^ = 8.451). Finally, the education level (*p* = 0.011, χ^2^ = 8.605), occupation (*p* = 0.002, χ^2^ = 18.335) and health centre of attachment (*p* < 0.0001, χ^2^ = 101.705) were significant in determining the knowledge of availability of screening services at the various health facilities.

**Conclusion:**

The following were found to significantly influence the knowledge of CHVs about cervical cancer: level of education, occupation, health facility of attachment and years of service as a CHV. There is need, therefore, for training on cervical cancer.

## Background

Globally, cervical cancer is the fourth most frequent cancer in women, with an estimated 530,000 new cases in 2012, representing 7.5% of all female cancer deaths. Of the estimated more than 270,000 deaths from cervical cancer every year, more than 85% of these occur in less developed regions. In developed countries, programmes are in place thnat enable women to be screened, making most pre-cancerous lesions identifiable at stages when they can easily be treated. Early treatment prevents up to 80% of cervical cancers in these countries [1]. In developing countries, cervical cancer is the second most common cancer, with an estimated 450,000 new cases in 2012 (84% of the new cases worldwide) [2]. Furthermore, it was estimated that 95% of women in developing countries had never been screened for cervical cancer, mainly due to lack of awareness amongst the population [1]. This underscores the need for public education that is undertaken by the community health volunteers (CHVs) under the community strategy arrangement.

In sub-Saharan Africa, 34.8 new cases of cervical cancer are diagnosed per 100,000 women annually, and 22.5/100,000 women die from the disease, making it the second most common cancer after breast cancer [3]. The disease burden is significantly higher in the developing countries with lower screening rates, largely due to lack of screening that allows for detection of a pre-cancerous lesion and early stage cervical cancer. Data from hospital-based registries in Kenya indicated that cancer of the cervix accounts for 70–80% of all cancers of the genital tract [4]. Despite the magnitude of the problem in Kenya and the fact that it is easily preventable, cervical cancer screening coverage in Kenya for all women aged 18 to 69 years is only 3.2% against a target of 70% coverage. In Kisumu County, only 2% of the women of reproductive age (WRA) were screened within the Kadibo Division, recording just a 1.5% screening rate in 2013 [5]. The Family AIDS Care and Education Services (FACES) programme, a local Non-Governmental Organization (NGO), was initiated and is supporting training and mentorship of healthcare workers in cervical cancer screening in various health facilities in Kisumu County [6], but the uptake of screening has been poor due to inadequate knowledge of the general population [7]. This trend requires public education to reverse and improve screening in order to identify those at risk of cervical cancer and to establish the prevalence of cervical cancer.

Furthermore, data from the 2014 Kenya Demographic and Health Survey (KDHS) indicates that only 14% of women aged 15–49 years have ever had a cervical exam [8]. The women, therefore, need to be enlightened about cervical cancer to create an enhanced need for screening. Use of CHVs has been shown to be effective in passing health information [9]; however, their ability to create demand for the screening services depends largely on their ability to pass correct information to community members. Under the community strategy approach adopted by the ministry of health of Kenya in 2006, the CHVs carry out public education on health-related issues, including cervical cancer screening and prevention, and refer community members to health facilities for the services. The reporting tool (MOH 514) also requires them to report on the number of clients referred for cervical cancer screening among other health indicators. This means that the CHVs create demand for the cervical cancer screening services in the community after training; however, information of their knowledge about cervical cancer was lacking.

Enlightened women who have access to information about their health and are able to make informed decisions have been shown to be more likely to seek cervical cancer screening [10, 11], while a high level of knowledge about cervical cancer was found to be a key predictor of screening intent [12]. To enhance cervical cancer screening and early detection, it is important that women access the most critical information, including the risk factors, signs and symptoms and where screening services can be accessed [13].

According to the World Health Organization (WHO), a risk factor is any attribute, characteristic or exposure of an individual that increases the likelihood of developing a disease or injury [1]. Some examples of the risk factors for cervical cancer are early onset of sexual activity, human papilloma virus (HPV) infection, smoking and immune suppression [14]. Prevention and control of disease and injury require information about the leading medical causes of illness and exposures or risk factors. This creates a focus on areas that can be changed or avoided rather than those that cannot be changed [15]. Those exposed to risk factors that cannot be changed can also seek early and regular check-ups. Knowledge about the risk factors is therefore a very important component of disease prevention and control. With the right information, the community members will be able to determine whether they are at risk or not and hence seek cervical cancer screening services accordingly. In Kadibo Division, even though information about the risk factors of cervical cancer is easily available to CHVs, information about the knowledge of risk factors of cervical cancer in women of reproductive age was lacking amongst them. As such, the current study assessed the knowledge among the community health volunteers of the risk factors associated with cervical cancer in Kadibo Division, Kisumu County, Kenya.

Disease manifestation is very important in its diagnosis, management and treatment [16]; therefore, health service providers must know the right signs and symptoms to look for in order to give the right and timely medical attention to any disease and curb the development and spread of the disease. The right information can also be passed to the public to enable them seek timely medical attention on noticing such signs [17–19]. The most appropriate channel to pass this information is through the community gatekeepers, the CHVs. Having the right information regarding signs and symptoms of cervical cancer in the community creates demand for the available screening services at the health facilities and enhances screening to establish the cervical cancer prevalence rate. The information regarding knowledge of the signs and symptoms of cervical cancer among community health volunteers in Kadibo Division, Kisumu County remains undetermined. Therefore, the current study set out to assess the knowledge of the signs and symptoms of cervical cancer among community health volunteers in Kadibo Division, Kisumu County.

The knowledge about the availability of medical services determines how the society embraces and utilizes such services [20]. It is important to have medical services available and accessible to the community in order to promote prevention, management and cure of medical conditions [21]. An informed community will create demand for the available screening services and in turn enable establishment of cervical cancer prevalence rate. This information on the available screening services, cost and duration of screening is usually passed to the community members by the CHVs. However, the information on the CHVs’ knowledge of the availability of cervical cancer screening services in Kadibo Division, Kisumu County remains unknown. As such, the current study assessed the knowledge of the availability of cervical cancer screening services among community health volunteers in Kadibo Division, Kisumu County.

Finally, the knowledge of an individual is influenced by various factors that act as a system of various specific issues to have a net effect on an individual’s general knowledge. These factors are collectively denoted as socio-demographic factors and include indicators such as gender, age, education level, religion, marital status and occupation. The effect of these socio-demographic factors on the knowledge of the CHVs about cervical cancer screening remained unknown in Kadibo Division, Kisumu County. As such, the current study determined the socio-demographic factors influencing the knowledge of the CHVs of cervical cancer.

## Method

### Study site

**Fig. 1:**
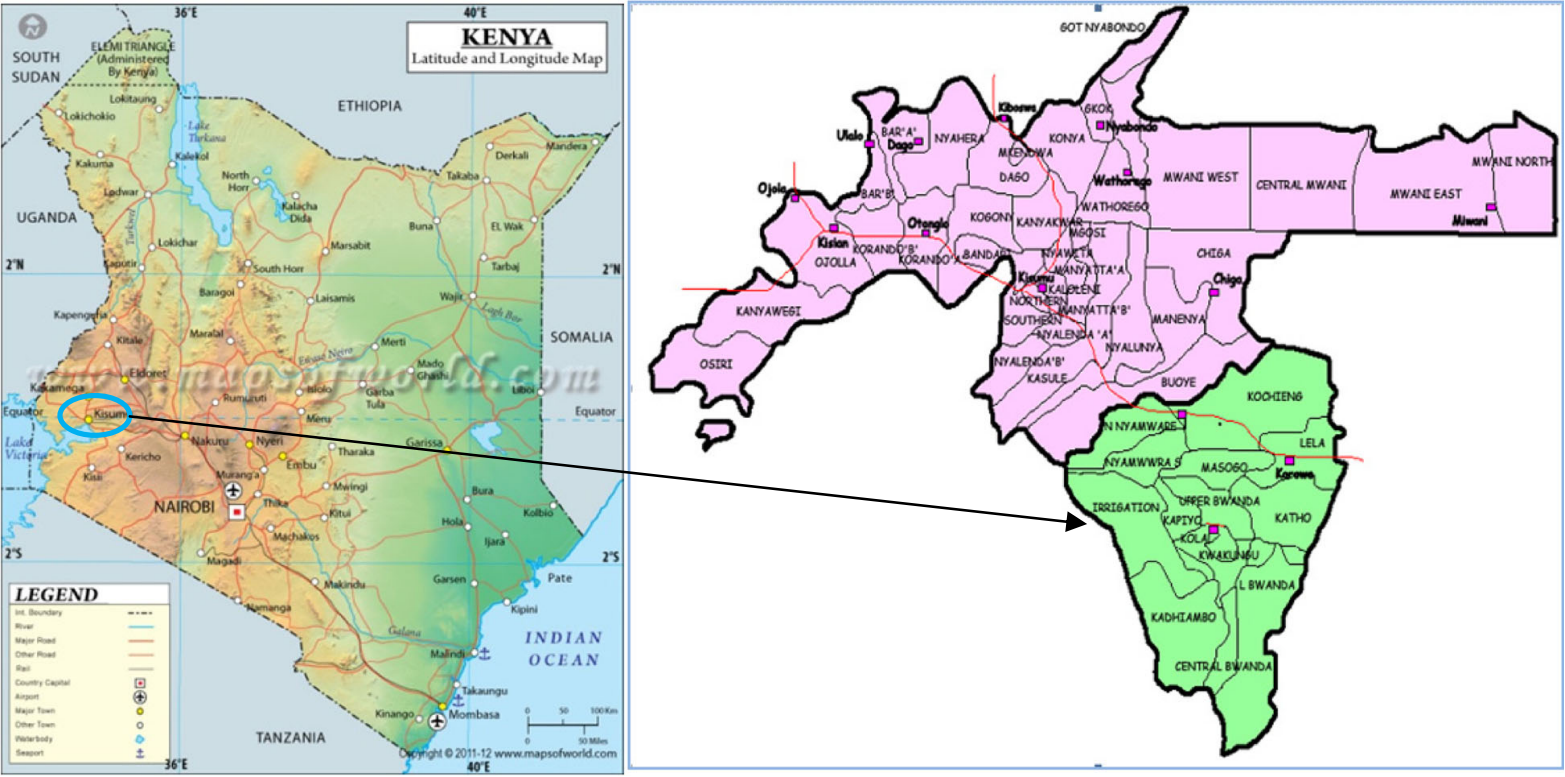
This is the map of the study site in Kadibo Division, Nyando Sub-County, Kisumu County, Kenya. It is served by four health centres, offering maternal and reproductive health services

The study was carried out in Kadibo Division, Kisumu County, which lies between latitudes −0.1959 and longitudes 34.8590 (Fig. 1: Study location). Kadibo has four government health facilities: namely, Rabuor, Nyangande, Kanyagwal and Hongo Ogosa. All these facilities offer maternal and child health services among other medical services. Each facility has community units attached to it, with each unit having 10 CHVs serving the households. The study site is in Nyando Sub-County, where, according to District Health Information Systems (DHIS) 2015, sexually transmitted infections accounted for 45.4% of the total cases attending special clinics in 2015. The prevalence of cervical cancer, however, remains unknown due to low screening rates, with only opportunistic screening being observed at the health facilities. The study site, though, boasts functioning community units with active CHVs, which made it ideal for this study.

### Target population

The targeted population was the registered CHVs attached to the government health facilities in Kadibo Division, Kisumu County. Their number, according to DHIS 2015 and the facility records, was 188. This was distributed as follows: 50 in Rabuor, 89 in Nyangande, 19 in Kanyagwal and 30 in Hongo Ogosa. All these CHVs were included in the current study.

### Study design

This was a cross-sectional saturated study in which all the 188 registered CHVs were interviewed about their knowledge regarding the risk factors, signs and symptoms and the availability of cervical cancer screening services. Since the required information could be collected through a one-time interview, the questionnaires for this study were administered to the CHVs only once.

### Sample size determination, sampling techniques and research instruments

Saturated sampling was done; therefore, all the targeted 188 CHVs were included in the study. The research instrument used was a semi-structured questionnaire.

### Data collection techniques

After obtaining written informed consent, data were collected through administration of the questionnaires to the study participants by trained research assistants.

### Data processing, presentation and analysis

Filled questionnaires were checked for completeness and correctness and then tallied and entered into an Excel spreadsheet before being exported to Statistical Package for Social Sciences (SPSS) for analysis. Participants’ demographic characteristics were presented by use of median and inter-quartile range for continuous variables and proportions and frequencies for categorical variables. Knowledge about cervical cancer was categorized based on percentage scores. There were three ordered categories of knowledge: low knowledge, average knowledge and high knowledge. Knowledge about signs and symptoms of cervical cancer was presented by use of frequencies and proportions. The knowledge about screening services at health facilities was presented by use of proportions. Chi-square testing was used to determine which demographic characteristics were important in determining the knowledge. *P* ≤ 0.05 were considered statistically significant.

### Ethical approval

The authority to carry out the study was obtained from the Maseno University’s School of Graduate Studies (SGS). Ethical approval was granted by the Maseno University Ethics Review Committee (MUERC) before recruitment into the study; the participants’ written informed consent was also sought. The authority of the Kisumu County Health Management was also obtained at the county, sub-county and facility levels. Lastly, the confidentiality of the information and the anonymity of the participants were guaranteed.

## Results

### Demographic characteristics of participants

The majority of the participants 161 (85.6%) were women, with only 27 (14.4%) being men. The distribution according to age was as follows: 3 for 20–24 years (1.6%), 13 for 25–29 years (6.9%), 30 for 30–34 years (16.0%), 26 for 35–39 years (13.8%), 37 for 40–44 years (19.7%), 30 for 45–49 years (16.0%), 26 for 50–54 years (13.8%), 13 for 55–59 years (6.9%) and 10 for 60–64 years (5.3%). A majority at 91 (48.4%) had a primary level of education, while 85 (45.2%) had a secondary level of education and only 12 (6.4%) respondents had a post-secondary level of education. All the 188 (100%) respondents were Christians. The majority of the respondents 168 (89.4%) were married, whereas 18 (9.6%) did not have spouses anymore (divorced, separated or widowed) with only 2 (1.1%) saying that they were single/never married. A majority of 132 (70.2%) were small-scale farmers, followed by 24 (12.8%) who were in business, 21 (11.2%) were manual labourers and 11 (5.9%) engaged in commercial farming. The respondents were able to indicate the duration over which they had worked as CHVs; a majority of the respondents, 91 (48.4%) in total, had worked for over 7 years, 70 (37.2%) had worked for 5–7 years, 24 (12.8%) had worked between 2 and 4 years and 3 (1.6%) had worked for less than 2 years, as presented in Table 1.

**Table 1:** Demographic Characteristics of the Study Participants

Respondent characteristics		Number of respondents
Gender	Male	27 (14.4%)
	Female	161(85.6%)
Age (Years)	20–24	3(1.6%)
	25–29	13(6.9%)
	30–34	30(16.0%)
	35–39	26(13.8%)
	40–44	37(19.7%)
	45–49	30(16.0%)
	50–54	26(13.8%)
	55–59	13(6.9%)
	60–64	10(5.3%)
Education level	Primary	91(48.4%)
	Secondary	85(45.2%)
	Post-secondary	12(6.4%)
Religion	Christian	188(100%)
Marital status	Single	2(1.1%)
	Married	168(89.4%)
	Separated	18(9.6%)
Occupation	Small scale farming	132(70.2%)
	Commercial farming	11(5.9%)
	Business	24(12.8%)
	Casual laborer	21(11.2%)
Facility of attachment	Rabuor	50(26.6%)
	Nyangande	89(47.3%)
	Hongo Ogosa	30(16.0%)
	Kanyagwal	19(10.1%)
Years of service	˂2	3(1.6%)
	2–4	24(12.8%)
	5–7	70(37.2%)
	˃7	91(48.4%)

Data are numbers (proportions)

### Knowledge about the risk factors associated with cervical cancer

The respondents were asked to list the risk factors associated with cervical cancer; they were allowed to give multiple responses that were then scored, and proportions were worked out given the number of risk factors that a respondent was able to list.

All 188 (100%) respondents indicated that they had heard about cervical cancer and correctly indicated that it affected women. It was established that the majority of the respondents at 128 (68.1%) had low knowledge of risk factors associated with cervical cancer, with only 60 (31.9%) recording average knowledge and none having high knowledge, as shown in Table 2. The mean score for the participants was calculated as 30.83% [minimum 0.00%, maximum 66.67%; standard deviation (SD) = 0.897], which was interpreted as having a generally low knowledge of risk factors associated with cervical cancer. The difference in the distributions of knowledge across the different categories was significant (*p* < 0.0001).

**Table 2:** Knowledge about the Risk Factors Associated With Cervical Cancer

	Many children	Many sexual partners	Early onset of sexual activity	HPV infection	Smoking	Immune suppression
Yes	7(3.7%)	130(69.1%)	99(52.7%)	100(53.2%)	3(1.6%)	9(4.8%)
No	181(96.3%)	58(30.9%)	89(47.3%)	88(46.8%)	185(98.4%)	179(95.2%)
Total	188(100%)	188(100%)	188(100%)	188(100%)	188(100%)	188(100%)
		Knowledge About Risk Factors				
		Low	Average	High		
Knowledge on risk factors		128(68.1%)	60(31.9%)	0(0.0%)		

Data are in numbers (percentage). Knowledge categories are based on the number of risk factors identified out of the six, converted to percentage then stratified as low, average and high knowledge

Having many sexual partners was identified by 130 (69.1%) respondents, HPV infection was identified by 100 (53.2%), and early onset of sexual activity was identified by 99 (52.7%). Immune suppression in 179 (95.2%), having many children in 181 (96.3%) and smoking in 185 (98.4%) were identified as non-risk factors for cervical cancer, as shown in Table 2.

The general knowledge about the risk factors associated with cervical cancer was affected by the level of education at (*p* = 0.012, χ^2^ = 3.839), occupation (*p* < 0.0001, χ^2^ = 12.722), and health centre of attachment (*p* < 0.0001, χ^2^ = 71.013). However, regression analyses showed that only the health facility of attachment was important in determining knowledge on risk factors associated with cervical cancer. For example, CHVs attached to Rabour were 70 times more likely to be knowledgeable [Odds Ratio (OR) =70.200, 95% Confidence Interval (CI) = 8.341–590.809, *p* < 0.0001] relative to other areas (Table 3).

**Table 3:** Relationship between Demographic Factors and Knowledge of Risk Factors Associated With Cervical Cancer

Demographic characteristic	Df	Many children (*p*-value)	Many sexual partners (p-value)	Early onset of sexual activity (*p*-value)	HPV infection (*p*-value)	Smoking (p-value)	Immune suppression (*p-*value)
Gender	1	0.269	0.229	0.356	0.272	0.475	0.096
Age	8	0.148	0.**003**	0.290	0.650	0.411	0.292
Education	2	0.465	0.517	0.728	0.310	0.800	**0.001**
Marital status	2	0.214	0.337	0.965	0.313	0.367	0.940
Occupation	3	0.093	**0.008**	**<0.0001**	0.270	0.374	0.617
Health centre	3	0.399	**<0.0001**	**<0.0001**	**<0.0001**	0.584	**0.001**
Duration worked	3	0.177	0.386	0.065	0.060	0.355	0.114
Demographic characteristics		Proportions	Knowledge on risk factors (*p* value)	Knowledge on risk factors (χ^2^ values)	Logistic regression		
					p-value	O.R. (95% CI)	
Gender	Male Female (Ref*)	NA*	0.929	0.520	0.472	1.402, (0.558–3.523)	
Age groups					0.089		
	20–24	NA*	0**.**142	5.518	0.287	0.167, (0.006–4.515)	
	25–29				0.022	0.050, (0.004–0.652)	
	30–34				0.162	0.183, (0.017–1.980)	
	35–39				0.121	0.155, (0.015–1.634)	
	40–44				0.071	0.111, (0.010–1.207)	
	45–49				0.103	0.136, (0.012–1.492)	
	50–54				0.531	0.458, (0.040–5.256)	
	55–59 (Ref*)						
Education level					0.150		
	Primary,	38.5%	**0.012**	3.839	0.731	1.250, (0.350–4.462)	
	Secondary,	24.7%			0.525	0.656, (0.179–2.402)	
	Post secondary (Ref*)	33.3%					
Marital status					0.418		
	Single	NA*	0.059	1.789	0.881	1.250, (0.067–23.259)	
	Married				0.228	0.545, (0.203–1.461)	
	Separated (Ref*)						
Occupation					0.086		
	Small scale,	39.4%	**<0.0001**	12.722	0.082	2.763, (0.880–8.670)	
	Commercial farming	0.0%			0.999	0.000	
	Business,	16.7%			0.835	0.850, (0.184–3.923)	
	Casual work (Ref*)	19.0%					
Health centre attached					**<0.0001**		
	Rabuor	80.0%,	**<0.0001**	71.013	**<0.0001**	70.200,(8.341–590.809)	
	Nyangande	16.9%			0.181	4.135, (0.516–33.150)	
	Hongo Ogosa	13.3%			0.541	2.077, (0.200–21.596)	
	Kanyagwal (Ref*)	5.3%					
Duration worked in years					0.673		
	<2	NA*	0.059	1.581	0.979	0.968, (0.084–11.095)	
	2–4				0.219	0.509, (0.174–1.494)	
	5–7				0.872	0.947, (0.489–1.834)	
	>7 (Ref*)						

Statistical significance determined by Chi-square and logistic regression analysis. Values in bold are statistically significant at *P* ≤ 0.05. Proportion represents participants with average knowledge. NA* not applicable. Ref* Reference

### Knowledge about the signs and symptoms of cervical cancer

The respondents were asked to list the signs and symptoms that are suggestive of cervical cancer, while allowing for multiple responses. The results were scored and proportions presented. Approximately 95 (50.5%) had low knowledge, 15 (8.0%) had average knowledge, and 78 (41.5%) had high knowledge. The mean score of the study participants was calculated as 58.75% (minimum 0%, maximum 100%, SD = 1.285). This was interpreted as average knowledge about signs and symptoms of cervical cancer amongst the CHVs. The difference in the distributions of the knowledge across the different categories was significant (*p* < 0.0001).

A majority of the respondents mentioned the following as the signs and symptoms of cervical cancer: abnormal vaginal bleeding by 114 (60.6%) respondents, abnormal vaginal discharge by 115 (61.2%) respondents, abdominal pain by 99 (52.7%) respondents and pain during sexual intercourse by 90 (47.9%) respondents. The results are shown in Table 4.

**Table 4:** Knowledge about the Signs and Symptoms of Cervical Cancer

	Abnormal vaginal bleeding	Abnormal vaginal discharge	Abdominal pains	Pain during sexual intercourse
Yes	114(60.6%)	115(61.2%)	99(52.7%)	90(47.9%)
No	74(39.4%)	73(38.8%)	89(47.3%)	98(52.1%)
Total	188(100%)	188(100%)	188(100%)	188(100%)
		Knowledge About Signs and Symptoms		
		Low	Average	High
Knowledge on signs and symptoms		95(50.5%)	15(8.0%)	78(41.5%)

Data are in numbers (proportions). Knowledge categorized based on the number of signs and symptoms identified out of the four, converted to percentage then stratified into low, average and high knowledge

Chi-square test results showed that there was no relationship between knowledge about signs and symptoms of cervical cancer and gender, age, education, marital status and the health centre of attachment of the respondent CHVs. Nonetheless, the occupation of the respondent was found to be significantly related to the knowledge of whether abdominal pain (*p* = 0.002) and pain during sex (*p* = 0.003) were signs and symptoms of cervical cancer, while years of work as a CHV was significantly related to knowledge of abnormal vaginal discharge as a sign or symptom of cervical cancer (*p* = 0.003).

Finally, the overall knowledge about the signs and symptoms of cervical cancer were determined by the occupation of the CHVs (*p* = 0.030, χ^2^ = 15.110) and the years of work as a CHV (*p* = 0.014, χ^2^ = 8.451), as shown in Table 5.

**Table 5:** Relationship between Demographic Factors and Knowledge of Signs and Symptoms of Cervical Cancer

Demographic characteristics	df	Abnormal vaginal bleeding (*p*-value)	Abnormal vaginal discharge (*p*-value)	Abdominal pains (*p*-value)	Pain during sex (*p*-value)
Gender	1	0.353	0.263	0.283	0.423
Age	8	0.189	0.787	**0.035**	1.114
Education	2	0.130	0.547	0.907	0.429
Marital status	2	0.824	0.203	0.292	0.495
Occupation	3	0.075	0.243	**0.002**	**0.003**
Health centre attached	1	0.263	0.283	0.928	0.423
Duration worked	3	0.725	**0.030**	0.941	0.915
Demographic characteristics		Proportions	Knowledge on signs and symptoms (*p* value)	Knowledge on signs and symptoms (χ^2^ values)	
Gender	Male, Female	NA*	0.263	0.171	
Age	20–24, 25–29, 30–34, 35–39, 40–44, 45–49, 50–54, 55–59, 60–64	NA*	0.239	14.175	
Education	Primary, Secondary, Post secondary	NA*	0.446	9.140	
Marital status	Single, Married, Separated	NA*	0.332	1.783	
Occupation	Small scale, Commercial farming, Business, Casual work	51.5% 9.1% 29.2% 28.6%	**0.030**	15.110	
Health centre attached	Rabuor, Nyangande, Hongo ogosa, Kanyagwal	NA*	0.060	86.472	
Duration worked in years	<2, 2–4, 5–7, >7	0.0% 41.7% 45.7% 44.4%	**0.014**	8.451	

Statistical significance determined by Chi-square analysis. Values in bold are statistically significant at *P* ≤ 0.05. Proportion represents participants with high knowledge. NA* not applicable

### Knowledge about the availability of screening services at the health facilities

The respondents answered questions on the various aspects of screening services offered at the health facilities with regards to the methods used, cost, the turn-around time and the rescreening interval. The proportion score for each respondent was determined and used to stratify their knowledge into the three categories. A majority at 77 (41.0%) had high knowledge, followed by low knowledge in 71 (37.8%), while 40 (21.2%) had average knowledge (Table 6). The mean score was found to be 59.27% (minimum 0%, maximum 86.67%, SD = 0.612). This was interpreted as average knowledge by the CHVs of the screening services for cervical cancer. The difference in the distributions of the knowledge was statistically significant (*p* < 0.0001).

**Table 6:** Knowledge about the Availability of Screening Services at the Health Facilities

Screening services components			Respondents	
Screening methods used	VIA		40(21.3%)	
	VILI		37(19.7%)	
	Pap smear		72(38.3%)	
	HPV testing		39(20.7%)	
Cost	Free		174(92.6%)	
	< Ksh. 100		3(1.6%)	
	>Ksh. 100		11(5.9%)	
Turn-around time	<30 Min.		69(36.7%)	
	30–60 Min		112(59.6%)	
	>60 Min		6(3.7%)	
Retesting interval	Semi-annually		92(48.9%)	
	Annually		90(47.9%)	
	Every 5 years		3(1.6%)	
	Over 5 years		3(1.6%)	
	Knowledge category			
	Low	Average	High	
Knowledge on the availability of screening services	71(37.8%)	40(21.2%)	77(41.0%)	

Data are in numbers (percentage). Knowledge categorized based on the amount of details about the screening services given, converted to percentage then stratified into low, average and high knowledge

Gender was significant in determining the knowledge about the cost of screening (*p* = 0.004), while age was important in determining knowledge on use of VILI as a screening method (0.003) and the TAT (*p* = 0.008). Education level was important in determining knowledge on use of VILI (*p* = 0.035) and VIA (*p* = 0.007) while occupation was significant in determining the knowledge about use of VIA (*p* = 0.004) and VILI (*p* = 0.008) and the TAT (*p* = 0.050). Finally, the health centre of attachment was important in determining knowledge about the use of VIA (*p* = 0.000) and VILI (*p* = 0.000), the cost of screening (*p* = 0.001), TAT (*p* = 0.001) and the frequency of screening (*p* = 0.000) while the duration of work was significant in determining the knowledge about use of VIA (*p* = 0.014), the cost of screening (*p* = 0.000), TAT (*p* = 0.018) and the frequency of screening (*p* = 0.003).

Education (*p* = 0.011) occupation (*p* = 0.002) and health centre of attachment (*p* < 0.0001) were significant in determining the knowledge levels on availability of screening services at the various health facilities. This is shown in Table 7.

**Table 7:** Relationship between Demographic Characteristics and Knowledge about Availability of Screening Services

Demographic characteristics	df	VIA (*p*-value)	VILI (*p*-value)	Cost of screening (*p*-value)	TAT (*p*-value)	Frequency of screening (*p*-value)
Gender	1	0.169	0.449	**0.004**	0.401	0.709
Age	8	0.148	**0.003**	0.820	**0.008**	0.604
Education	2	**0.035**	**0.007**	0.494	0.402	0.374
Marital status	2	0.324	0.447	0.253	0.161	0.984
Occupation	3	**0.004**	**0.008**	0.088	**0.050**	0.108
Health centre attached	1	**<0.0001**	**<0.0001**	**0.001**	**0.001**	**<0.0001**
Duration worked	3	**0.014**	0.495	**<0.0001**	**0.018**	**0.003**
Demographic characteristics			Proportions	Knowledge on availability of screening services (*p* value)	Knowledge on availability of screening services (χ^2^ values)	
Gender	Male, Female		NA*	0.283	19.792	
Age	20–24, 25–29, 30–34, 35–39, 40–44, 45–49, 50–54, 55–59, 60–64		NA*	0.055	14.711	
Education	Primary, Secondary, Post secondary		8.0% 7.4% 1.1%	**0.011**	8.605	
Marital status	Single, Married, Separated		NA*	0.292	4.470	
Occupation	Small scale, Commercial farming, Business, Casual work		18.9% 0.0% 1.1% 19.0%	**0.002**	18.335	
Health centre attached	Rabuor, Nyangande, Hongo ogosa, Kanyagwal		28.0% 3.4% 13.3% 57.9%	**<0.0001**	101.705	
Duration worked in years	<2, 2–4, 5–7, >7		NA*	0.271	6.558	

Statistical significance determined by Chi-square analysis. Values in bold are statistically significant at *P* ≤ 0.05. Proportion represents participants with high knowledge. NA* not applicable

It was further established that all 188 (100.0%) of the respondents knew that screening services were available at the health facilities where they were attached; however, they did not have the right information with respect to the screening methods used, with only 40 (21.3%) and 37 (19.7%) respondents correctly identifying visual inspection using acetic acid (VIA) and visual inspection using Lugol’s iodine (VILI) as the methods being used, respectively. A majority of 174 (92.6%) correctly mentioned that the services were being offered free of charge; however, the turn-around time for the screening tests and the re-testing interval were only known by 69 (36.7%) and 3 (1.6%), respectively (Table 6).

### Socio-demographic factors affecting knowledge about cervical cancer

The knowledge about the risk factors associated with cervical cancer was determined by education (*p* = 0.012, χ^2^ = 3.839), occupation (*p* < 0.0001, χ^2^ = 12.722), and health centre of attachment (*p* < 0.0001, χ^2^ = 71.013). However, the knowledge of the signs and symptoms of cervical cancer was determined by the occupation of the CHVs (*p* = 0.030, χ^2^ = 15.110) and the years of work as a CHV (*p* = 0.014, χ^2^ = 8.451), while education (*p* = 0.011, χ^2^ = 8.605), occupation (*p* = 0.002, χ^2^ = 18.335) and health centre of attachment (*p* < 0.0001, χ^2^ = 101.705) were significant in determining the knowledge of availability of screening services at the various health facilities.

## Discussion

### Knowledge of the risk factors associated with cervical cancer

This study generally established that there was low knowledge of the risk factors of cervical cancer. This was in agreement with the findings of other studies [14] that established that Vietnamese American women were unable to correctly identify the cervical cancer risk factors. Another study [22] in Uganda noted that the knowledge level among medical workers was low at less than 40%, while another [23] determined that the awareness of human papillomavirus (HPV) as a risk factor for cervical cancer was at a very low proportion of 2.5%. In addition, other studies [24, 25] also demonstrated low knowledge of risk factors amongst the women in Wielkopolska region and Ethiopian health care workers.

This low knowledge can be attributed to the generally low education levels, which also have a bearing on the occupation of the CHVs. Lack of uniform training for the CHVs on the risk factors associated with cervical cancer and the fact that cervical cancer screening has also been neglected even though it is an important component of maternal health also contributed to the low knowledge. There is a need, therefore, to teach CHVs the risk factors of cervical cancer, since through them, the information can be passed to the community.

### Knowledge on the Signs and Symptoms of Cervical Cancer

There was great disparity in the knowledge about the signs and symptoms, with an average of 95 (50.5%) having low knowledge while 78 (41.5%) had high knowledge and only 15 (8.0%) had average knowledge.

These findings diverge with those from earlier studies [26] that pointed to low knowledge of warning signs/symptoms of cervical cancer amongst study participants in the upper mid-western states. One study [27] established that the knowledge of the signs and symptoms of cervical cancer was as low as 6.3% amongst participants in North Bengal, India, while another study [28] observed a lack of knowledge of the signs and symptoms of cervical cancer amongst students at a medical school in Al-Ahsa, Kingdom of Saudi Arabia.

The average knowledge observed could be due to the ongoing sensitization in the audio-visual media, which mainly focuses on passing of information on the signs and symptoms to look out for but neglects other aspects. However, the disparity between the high and low knowledge needs to be investigated.

### Knowledge of the availability of screening services

The knowledge of the details of the screening services ranged from low to high, with 71 (37.8%) of the respondents having low, 40 (21.2%) having average and 77 (41.0%) having high knowledge.

It was established that only 40 (21.3%) and 37 (19.7%) of the respondents were able to correctly identify VIA and VILI as the screening methods available in the health facilities. A majority of 71 (37.8%) wrongly identified Pap smear, followed by HPV testing by 40 (21.3%), as being available in the health facilities. A majority of the respondents (176 (93.6%)) across the health facilities mentioned that the screening services were available free of charge; 10 (5.3%) of the respondents mentioned that the screening services cost more than Ksh.100, while a minority of 2 (1.1%) indicated that the cost of screening was less than Ksh.100. The screening services take less than 30 min, as correctly mentioned by 71 (37.8%) of the respondents; however, a majority of 111 (59.0%) thought the screening takes 30–60 min, while 6 (2%) said that screening takes more than 60 min. A majority of the respondents at 92 (48.9%) said routine screening should be done semi-annually, and 90 (47.9%) said annually, with only 3 (1.6%) saying every 5 years and the same for after 5 years. In fact, routine screening should be once every five years.

It was evident that a majority of the CHVs were not aware of the screening methods available and the duration of screening. This was consistent with the findings of [29], where the purpose and importance of a Pap smear were not well understood amongst the high-risk women studied. Another study [30] established that 3% of female health workers did not know about the availability of the services and therefore did not seek screening and were therefore likely to pass wrong information to the community. Furthermore, in previous studies [31–33], it was established that a lack of awareness of availability of screening centres locally, cost and time were the main reasons given by respondents for not being screened.

The low score on the knowledge of methods of screening, the duration of screening and retesting interval were found to be low due to lack of training for the CHVs on the details of the screening services available. However, the high score on cost can be explained by the fact that all the services at the government health centres and dispensaries are currently free of charge. There was a significant relationship between the centre a particular CHV is attached to and the screening method available in the facility (*p*˂0.001).

The general knowledge was significantly related to education levels, which also influences occupation. These factors had a bearing on the kind of information a person vests their interest in. This average knowledge on availability of cervical cancer screening services is not sufficient for the gatekeeper role played by CHVs, and therefore, there is a need for their training and sensitization to pass accurate and consistent information to the community.

### Socio-demographic factors affecting the knowledge of cervical cancer

Level of education (*p* = 0.012), occupation (*p* < 0.0001), and facility of attachment (*p* < 0.0001) were found to significantly affect knowledge about risk factors associated with cervical cancer. This was similar to previous findings [34–36] that observed level of education and occupation to significantly affect knowledge of cervical cancer. CHVs with just a primary level education had better knowledge about the risk factors associated with cervical cancer than those with higher education. This is an indication that the formal education does not give more information about cervical cancer risk factors; therefore, there is a need for more training of the CHVs. The CHVs involved in small-scale farming were also found to have better knowledge. Lack of uniform training for the CHVs through health talks and continuous medical education on the risk factors associated with cervical cancer in the various health facilities explains why the health facility of attachment was a significant determinant of the knowledge of CHVs about cervical cancer. Rabuor had the most consistent series of health talks, and this has an effect on the scores obtained by the CHVs attached to these centres.

Occupation (*p* = 0.030) and duration of service as a CHV (*p* = 0.014) were found to be significant in determining the knowledge about signs and symptoms of cervical cancer as was also demonstrated in previous studies [25, 36, 37]. CHVs who practise small-scale farming were found to be more knowledgeable, similarly to those who had served for more than 5 years as CHVs. With more years of service, the CHVs gain experience and more information from their interaction with the health care workers and within themselves.

The knowledge of availability of screening services was significantly related to the level of education (*p* = 0.011), similar to previous findings [38, 39]. The CHVs with post-secondary education were found to have better knowledge. This could allude to the complexity of the details of the screening methods that require more brain power to comprehend. Occupation (*p* = 0.002) was also significant in determining knowledge of availability of cervical cancer screening services. This was similar to earlier findings [25, 36, 39], which also found occupation to significantly affect knowledge of cervical screening. Those CHVs practising small-scale farming and casual labourers were found to be more knowledgeable. Occupation generally has a bearing on the kind of information a person vests interest in. Finally, the health facility of attachment (*p* < 0.0001) was also significant in determining the knowledge about the availability of cervical cancer screening services in the respective health facilities. CHVs attached to Kanyagwal were also knowledgeable about screening services since because of their small number, they have more contact with the technical staff offering the services and thus better exposure to such information.

## Conclusions

The community health volunteers had low knowledge of the risk factors associated with cervical cancer. There was notable misinformation among the CHVs as far as risk factors for cervical cancer are concerned. The CHVs had an average knowledge about the signs and symptoms of cervical cancer; however, some signs and symptoms were not known by the majority of the respondents. Being the community gatekeepers, average knowledge is not sufficient to pass to the community since it will result in mis-informed populations. There is a need, therefore, to enlighten the CHVs on the signs and symptoms of cervical cancer. There was also average knowledge about the screening services that were available in the health facilities. The CHVs had inaccurate information with regard to the availability of the various screening methods at the facilities, how long it takes to have screening done and the rescreening interval. Lastly, the following were found to significantly influence the knowledge of CHVs about cervical cancer: level of education, occupation, health facility of attachment and years of service as a CHV.

## Abbreviations

ACCP: Alliance for Cervical Cancer Prevention
AIDS: Acquired immunodeficiency syndrome
CCS: Cervical cancer screening
CHV: Community health volunteer
CI: Confidence interval
DHIS: District health information systems
FACES: Family Aids Care and Educational Services
HIV: Human immunodeficiency virus
HPV: Human papillomavirus
IARC: International Agency for Research on Cancer
ICC: Invasive cervical cancer
KNH: Kenyatta National Hospital
NGO: Non-governmental organisation
OR: Odds ratio
SD: Standard deviation
SGS: School of graduate studies
STI: Sexually transmitted infection
TAT: Turn-around time
VIA: Visual inspection with acetic acid
VILI: Visual inspection using Lugol’s Iodine
WHO: World Health Organization
WRA: Women of Reproductive Age
